# Annotations

**Published:** 1909-10-09

**Authors:** 


### Annotations.

### The Late Sir Thomas Smith.

We have to record the death, at the age of 76, of Sir Thomas Smith, Bt., K.C.V.O., F.R.C.S., the Honorary Serjeant Surgeon to the King, and Consulting Surgeon to St. Bartholomew's Hospital. It is not only in his public capacity that the loss of Sir Thomas will be felt. He had a wide circle of friends, many of them his pupils, during his long connection in so many capacities with St. Bartholomew's Hospital, which he served so well. Those who heard him lecture or met him in private life have many memories of his genial conversation and quaint humour. He was always a good friend to medical students, and from the time he took his F.R.C.S. in 1858 kept up an active interest in their life and work. Born in March, 1833, the son of Mr. Benjamin Smith, of Great Lodge, Kent, he began as a young man that study of medicine, which, among a host of distinctions, gave him later the honour of being Vice-President of the Royal College of Surgeons of England. Besides being Consulting Surgeon to "Bart's," the Children's Hospital, Great Ormond Street, the Alexandra Hospital for Hip Disease, and King Edward VII.'s Hospital for Officers, he had been also Examiner in Surgery to the Royal College of Physicians. Many contributions to the medical newspapers came from his pen, and he added to his medical interests a keen activity in the Corporation of the Sons of the Clergy, being a member of its Court of Assistants, and having sat on its petitions committee. Few men in the profession had a wider general experience of medicine and hospital matters than he, who made his home in the centre of hospital life, and permeated it with his natural kindness of heart as much as with his scientific and practical ability. He was, in fact, one of the fathers of the profession who, wherever he touched it, has left a host of grateful memories behind him.

### Ptomaine Poisoning and Food Preservatives.

In an interesting paper read before the Indiana State Pharmaceutical Society, Dr. Harry Barnard, the State Food and Drug Commisioner, discussed the preservation of syrups and crushed fruits, and incidentally touched upon the important question of ptomaine poisoning. One of the main arguments for the justification of the addition to foodstuffs of so-called "harmless preservatives" is found in the alleged fact that ptomaine poisoning is unknown with chemically preserved food. It is argued that ordinary foodstuffs are liable to deteriorate, and when eaten in that state to induce symptoms of gastro-intestinal disturbance. Preservatives are thus added in perfect good faith, but as Dr. Barnard rightly points out, the argument is perfectly invalid. Dealing more especially with fruits and fruit syrups that are so largely used in America in the preparation of those popular concoctions dispensed at the "soda fountain," he remarks: "There never yet has been a case of ptomaine poisoning or any other poisoning, aside from that produced by the injection of a greater amount of food than the stomach can care for, following the eating of crushed fruits and fruit syrups dispensed at the soda fountain in bad condition. Ptomaine poisoning can never follow the eating of such fruits, since ptomaines are produced only by the decomposition of albuminoid and proteid material and not by the decomposition of sugar. Crushed fruits and fruit syrups spoil because they ferment and thus change somewhat their character; but will anyone maintain that the process of fermentation is injurious to the health? Is fermented grape juice full of ptomaines and other poisons? Is cider, which is fermented apple juice, injurious to health? When your crushed fruits go bad or your fruit syrups spoil they do so because of the usual forms of fermentation which turn sugar into alcohol and alcohol into acetic acid are taking place, and goods in this condition are not one whit less wholesome than unfermented if we do not take into consideration the traces of alcohol which are always present." With these conclusions every practitioner who has had any experience of gastro-intestinal symptoms arising in summer-time from the ingestion of fruitarian drinks and dishes must wholly agree. The addition of preservatives to food usually hides decomposition, but does not always prevent it.

### Hospital Congresses.

The recent session of the American Hospital Congress draws attention to the good work that may be done by those interested in charitable institutions and their welfare by occasionally gathering in common council and discussing the affairs which affect them. It is not only in America that such a congress should exist or where it would prove useful. With us, too, it might prove of the greatest benefit to all our charitable organisations and institutions by offering a platform on which mutual interests would be discussed with less selfishness and one-sidedness than can be exhibited in the ordinary board-rooms. Such a congress to be successful should be a huge board of directors, an advisory council as it were to the hospital world. The Hospitals Association, we are glad to hear, has taken up this question and will deal with it practically this autumn. A hospital congress on the American plan held annually or biennially, the place of meeting being carefully chosen each year, so as ultimately to include all the great centres of population, could not fail to be both popular and helpful to all interested in the hospitals. On a question like that of the establishment of independent and self-contained school clinics, for instance, all the parties should speak, though not at the same time nor without due regard to the interests of those outside each one's individual sphere of influence. We venture to think that a periodical gathering of hospital managers, committee-men, honorary staff, and chairmen would have a stimulating influence upon hospital work and thought in this country. It would therefore subserve the highest interests of the community at large by bringing the public into closer touch with the many problems that confront hospital administrators.

